# Supplementary figures and images for: Revisiting bicoid function: complete inactivation reveals an additional fundamental role in Drosophila egg geometry specification
Source: Hereditas. 2024 Jan 2;161:1. doi: 10.1186/s41065-023-00305-9 (PMC10759373; doi:10.1186/s41065-023-00305-9)

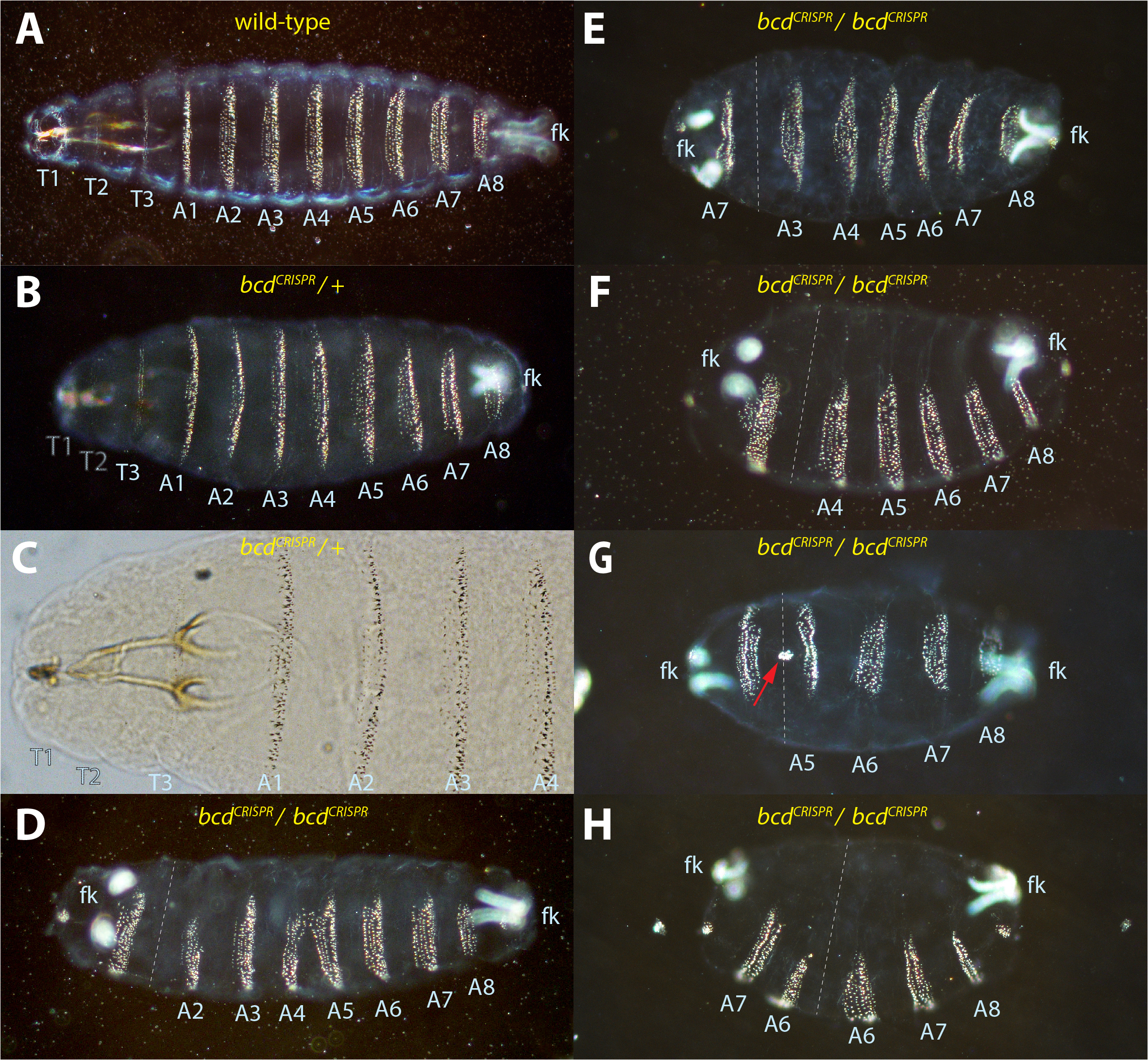

Supplement: Supplementary file 1 — Supplementary material 1:Fig. S1 Phenotypic appearance of bcdCRISPR mutants. (A) cuticle of a wild-type larva as reference. (B) cuticle of a larva from heterozygous bcdCRISPR mothers and (C) anterior tip of the embryo in (B) to highlight the weak head phenotype with lack of cuticular patterning of T1 and T2. (D-H) cuticles from homozygous bcdCRISPR mothers, illustrating the phenotypic variability, with (G) representing the highest percentage for the phenotypic appearance. (D) cuticle where A2-A8 is present with a small posterior end of unknown segmental identity and the filzkörper (fk) duplicated to the anterior. (E) cuticle where A3-A8 is present with a small duplicated posterior end, A7 and the filzkörper. (F) cuticle where A4-A8 is present with a duplicated posterior end with unknown segmental identity and the filzkörper. (G) cuticle where A5-A8 is present with a duplicated posterior end, A7 and the filzkörper. (H) cuticle where A6-A8 is present with a duplicated posterior end, A7 and A6, and the filzkörper. Red arrow points towards a small circle of denticles infrequently observed, as also shown in Fig. 2L. All cuticles are oriented anterior to the left and showing the ventral side, unless otherwise noted. Identities of segments are indicated as abbreviations wherever identification was possible. [file 41065_2023_305_MOESM1_ESM.jpg]
